# Risk of venous thromboembolism in association with factor V leiden in cancer patients – The EDITH case-control study

**DOI:** 10.1371/journal.pone.0194973

**Published:** 2018-05-18

**Authors:** Adeline Heraudeau, Aurélien Delluc, Mickaël Le Henaff, Karine Lacut, Christophe Leroyer, Benoit Desrues, Francis Couturaud, Cécile Tromeur

**Affiliations:** 1 Service de pneumologie, Hôpital Pontchaillou, Université de Rennes 1, Rue Henri-Le-Guilloux, France; 2 Université de Brest, EA3878 (GETBO) IFR, Brest, France; 3 Département de médecine interne et pneumologie, CHRU de la Cavale Blanche, Boulevard Tanguy Prigent, France; 4 Service de pneumologie, Hôpital du Scorff, Avenue de Choiseul, France; 5 INSERM, CIC, Brest, France; 6 INSERM, ERL, Rennes, France; Institut d'Investigacions Biomediques de Barcelona, SPAIN

## Abstract

**Background:**

Cancer and factor V Leiden mutation are both risk factors for venous thromboembolism (VTE). Cancer critically increases the thrombotic risk whereas Factor V Leiden is the most common pro-thrombotic mutation. The impact of the factor V Leiden on the risk of VTE in cancer patients remains uncertain.

**Objective:**

To assess the impact of factor V Leiden mutation in cancer-associated thrombosis.

**Methods:**

The EDITH hospital-based case-control study enrolled 182 patients with cancer and VTE as well as 182 control patients with cancer, matched for gender, age and cancer location, between 2000 and 2012, in the University Hospital of Brest. All cases and controls were genotyped for the factor V Leiden mutation and interviewed with a standardized questionnaire.

**Results:**

Twenty one of 182 (11.5%) patients with cancer-associated thrombosis carried the factor V Leiden mutation and 4 of 182 (2.2%) controls with cancer but no venous thrombosis. In multivariate analysis including cancer stage and family history of VTE, cancer patients with factor V Leiden mutation had a seven-fold increased risk of venous thromboembolism (adjusted odds ratio [OR], 7.04; 95% CI, 2.01–24.63).

**Conclusion:**

The pro-thrombotic Factor V Leiden mutation was found to be an independent additional risk factor for venous thromboembolism in cancer patients and might therefore be considered in the individual thrombotic risk assessment.

## Introduction

Venous thromboembolism (VTE) is a frequent multifactorial disease defined as the occurrence of deep vein thrombosis (DVT) and/or pulmonary embolism (PE). Risk factors for VTE are well known, either acquired such as pregnancy, surgery and active malignancy, or inherited such as pro-thrombotic mutations [1].

VTE is associated with a worse prognosis in cancer patients and the second cause of death in this population [2,3]. Cancer is associated with a four- to seven-fold increased risk of VTE [4,5]. The magnitude of this risk varies depending on tumor site, stage, histology and antineoplastic treatment such as surgery, chemotherapy or hormonotherapy [6]. The risk of VTE-related death is also critically increased in cancer patients [7], due to thrombosis recurrences after stopping anticoagulation and due to bleeding under anticoagulant therapy [8].

Among genetic risk factors for VTE [9–11], factor V Leiden (FVL) mutation, is the most common with a prevalence of about 5% in the Caucasian population, followed by the G20210A prothrombin gene mutation that affects 2% of Caucasians [9,10]. The FVL mutation is mostly caused by a single R506Q mutation in the factor V gene and leads to a three- to five- fold increased risk of first VTE event in heterozygous carriers [12]. In presence of the G20210A prothrombin gene mutation, the risk of VTE is increased about two- to three-fold [12].

The role of FVL mutation in cancer-associated thrombosis remains unclear, with only a few studies showing various results. The only large prospective study, to our knowledge, performed on an observational Austrian cohort of nearly one thousand cancer patients, showed a two-fold increased risk of VTE associated with the FVL mutation with 13.9% of subjects with FVL developing VTE [13]. Similarly, a large case-control study found a higher VTE risk in patients with both cancer and FVL mutation (Odds ratio [OR] of 12) compared with cancer alone (OR of 7) [5]. However, another case-control study and a small prospective trial failed to demonstrate an increased risk of cancer-associated thrombosis in association with FV Leiden mutation [14,15]. Lastly, a synergistic effect between cancer and factor V Leiden on VTE risk was reported in one large case-cohort study [16].

Based on the E.D.I.T.H. case control study, we aimed to determine if there was an association between FVL mutation and the risk of VTE in patients with cancer.

## Methods

### Study design

The observational EDITH case-control study was designed to assess interactions between genetic and environmental risk factors for VTE. Between May 2000 and November 2011, all consecutive objectively proven VTE cases diagnosed in Brest University Hospital were included in a registry. Methods have been extensively detailed previously [17].

### Study population

Selection of cases: all patients admitted to hospital or seen at the outpatient clinic with an objectively proven symptomatic VTE during the study period and a cancer (i.e.; active cancer or cancer not resolved in the past two years) were eligible for inclusion. DVT of the lower limbs either distal or proximal (involving the popliteal vein or above) had to be confirmed by real time B mode compression ultrasonography (CUS). The diagnostic criteria for symptomatic PE were either: (1) a high clinical probability and a high-probability ventilation-perfusion lung scan according to the PIOPED [18] criteria, or (2) a proximal DVT shown by ultrasonography in a patient with symptoms of pulmonary embolism, or (3) a positive computed tomography pulmonary angiography (CTPA scan) with a central filling defect outlined by contrast material or a complete occlusion in a segmental or more proximal pulmonary artery. All DVT and PE patients had a complete ultrasonography of the lower limbs and all VTE events were validated by three physicians (AD, KL, FC). Exclusion criteria were the following: (1) age under 18 years, (2) residence outside Brest district or the nearby three other districts, (3) inability to sign a written informed consent and, (4) patient refusal. All patients with a symptomatic VTE associated to an active malignancy who participated to the EDITH study between May 2000 and November 2011 were included in this analysis.

Selection of controls: for each case, one control was selected among patients hospitalized in the same ward or seen at the outpatient clinic. Controls and cases were matched by age (within a ten-year age band), gender and cancer site. Cases’ exclusion criteria applied to controls, with in addition exclusion of subjects receiving lifelong anticoagulant therapy or having a history of previous objectively documented VTE.

The EDITH protocol was approved by our hospital scientific and ethic board (Person Protection Committee Ouest 6). A specific written informed consent for participation in the study and for DNA analysis was obtained for all case and control patients.

### Data collection

Cases and controls were interviewed during their hospital stay through a one-to-one standardized questionnaire. Demographic data, general characteristics, medication use as well as risk factors for VTE were collected and patients’ files were checked for relevant informations.

### Laboratory analysis

Blood was collected in 0.05 M EDTA for DNA analysis. Genomic DNA was extracted from peripheral leukocytes from EDTA-anti-coagulated blood using a commercially available DNA isolation kit. Genotype for the factor V Leiden gene’s mutation (R506Q mutation) was determined as previously described [19,20].

### Statistical analysis

Assuming a frequency of 5% of FVL mutations in the control group, we calculated that 300 patients will be required to have a 90% power to detect a 4-fold increased VTE risk associated with FVL mutation, with a two-sided alpha level of 5% and a 1:1 ratio. Patients’ characteristics were compared using Wilcoxon paired test or McNemar Chi-square test when appropriate. Conditional logistic regression was carried out to estimate OR and 95% confidence intervals (CI) as measurement of the association between VTE risk and FVL mutation in cancer patients. Any variable having a p-value of 0.1 or less in the paired analysis was included in the multivariate conditional logistic regression model. Statistical analysis used procedures available in STATA software, version 14.1 (StataCorp, College Station, TX, USA).

## Results

### Patient characteristics

**Fig 1:**
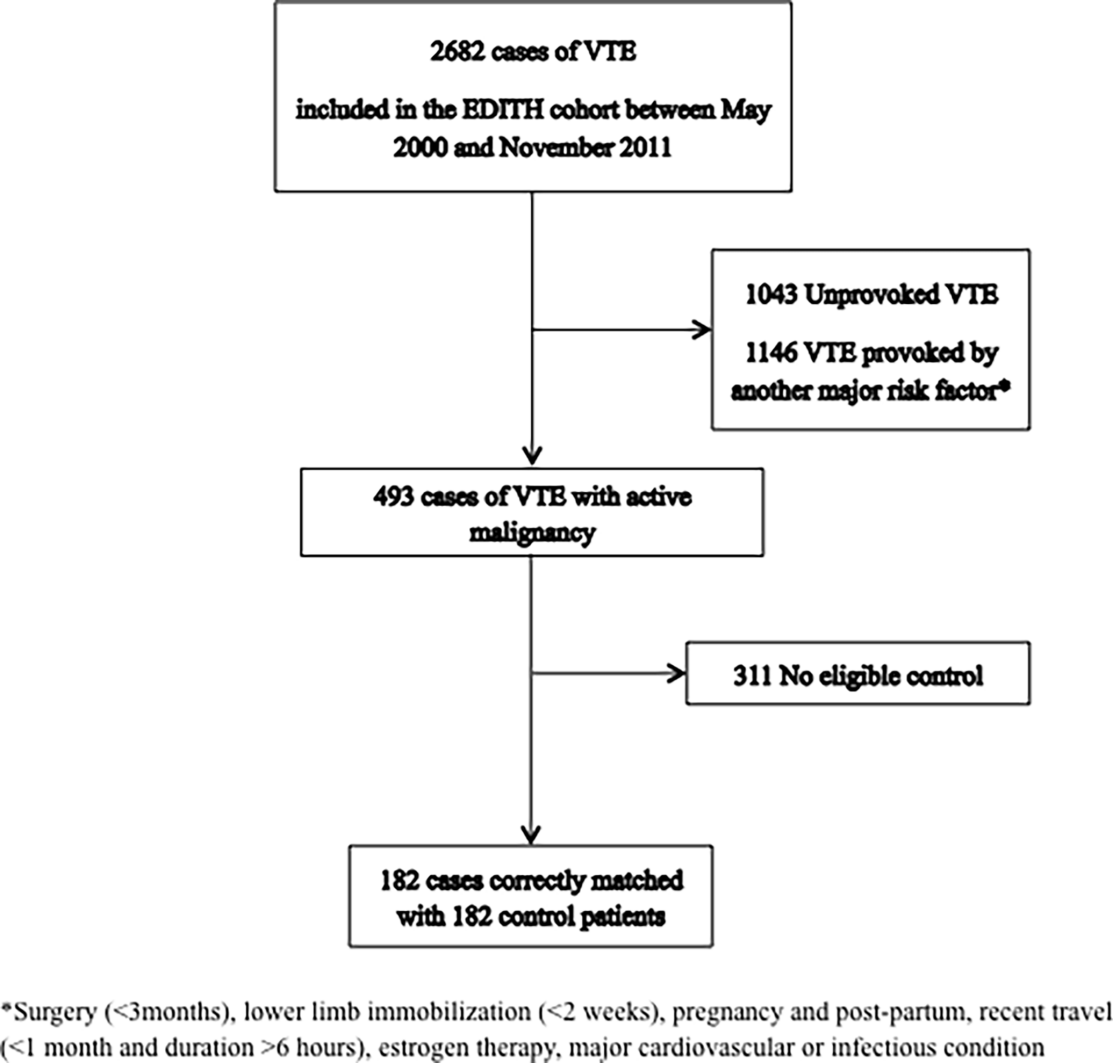
Flow chart.

Between May 2000 and November 2011, among 2682 cases of VTE enrolled in the EDITH cohort study in Brest University Hospital, 1043 patients had an episode of unprovoked venous thrombosis, 493 patients had an active malignancy and 1146 were provoked by risk factors other than cancer. Finally, 182 cancer-associated thrombosis cases were properly matched with 182 control subjects included until November 2012 (Fig 1).

Detailed participant characteristics of the 364 patients included are given in Table 1. The median age was 70 (Interquartile range [IQR] 61–77) for cases and not different from the controls. A family history of VTE tended to be more frequent in cases (25 of 182 [13.7%]) than in controls (14 of 182 [7.7%]) (p = 0.08). There was a personal past history of VTE for 33 cases (18.1%) but none for controls (exclusion criteria) (p<0.0001). Most of the cases had symptomatic PE associated with DVT (97/182 cases).

**Table 1:** Patients characteristics.

Parameters	Cases N = 182	Controls N = 182	P-value*
**Age** (years), median [IQR]	70 [61–77]	69 [62–78]	0.13
**Gender, male**, n (%)	115 (63.2%)	115 (63.2%)	1
**Body Mass Index** (kg/m^2^), mean [SD]	25.3 ± 5.2	24.7 ± 4.8	0.13
**Cancer Stage**, n (%)			
Metastatic disease	83/172 (48.3%)	78/177 (44.1%)	0.08
No distant metastasis	71/172 (41.3%)	86/177 (48.6%)	
Not classifiable	18/172 (10.5%)	13/177 (7.3%)	
**VTE location**, n (%)			
PE ± DVT	97/182 (53.3%)	**NA**	
Isolated proximal DVT	68/182 (37.4%)		
Isolated distal VTE	17/182 (9.3%)		
**Past history of VTE**, n (%)	33/182 (18.1%)	0	<0.0001
**Family history of VTE**, n (%)	25/182 (13.7%)	14/182 (7.7%)	0.08
**Time from cancer diagnosis to inclusion into the study** (days), median [IQR]	341 [93–1316]	263 [69–1319]	0.43
**Site of cancer**, n (%)			
Lung	37/182 (20.3%)	36/182 (19.8%)	0.54
Prostate	35/182 (19.2%)	37/182 (20.3%)	
Breast	20/182 (11.0%)	17/182 (9.3%)	
Colorectal	13/182 (7.1%)	16/182 (8.8%)	
Pancreas	13/182 (7.1%)	10/182 (5.5%)	
Esophagus and stomach	9/182 (4.9%)	8/182 (4.4%)	
Lymphoma	8/182 (4.4%)	8/182 (4.4%)	
Other hematologic	18/182 (9.9%)	15/182 (8.2%)	
Other genito-urinary	17/182 (9.3%)	19/182 (10.4%)	
Other	12/182 (6.6%)	16/182 (8.8%)	
**Histology**, n (%)			
Adenocarcinoma	108/169 (63.9%)	106/176 (60.2%)	0.26
Solid tumor of other histology	35/169 (20.7%)	48/176 (27.3%)	
Lymphoma	8/169 (4.7%)	9/176 (5.1%)	
Other hematological malignancy	18/169 (10.6%)	13/176 (7.4%)	
**Treatment**, n (%)			
Surgery	79/154 (51.3%)	82/156 (52.6%)	0.65
Chemotherapy	94/149 (63.1%)	90/148 (60.8%)	0.66
Radiotherapy	45/150 (30.0%)	46/151 (30.5%)	1
**Factor V Leiden mutation**, n (%)	21/182 (11.5%)	4/182 (2.2%)	0.0004
**G20210A prothrombin gene mutation**, n (%)	5/182 (2.7%)	1/182 (0.5%)	0.23

Data are given as number of patients (n) and percent (%).

IQR, interquartile range; SD, standard deviation; VTE, venous thromboembolism; PE, pulmonary embolism; DVT, deep vein thrombosis

* For the following variables, « Cancer Stage », « Time from cancer diagnosis to inclusion into the study », « Histology », « Surgery », « Chemotherapy », « Radiotherapy », the number of matched case-control pairs was 168, 172, 163, 133, 122 and 125, respectively

Regarding to cancer stage, 71 of 172 (41.3%) cases had no distant metastasis versus 86 of 177 (48.6%) controls (p = 0.08). The sites of cancer were similar for cases and controls: the most frequent were lung, prostate and breast, respectively with 20.3%, 19.2% and 11.0% each in the cases population. The G20210A prothrombin gene mutation was found in 5 cases and 1 control (2.7% vs. 0.5%, p = 0.23). All other characteristics including histology and treatment modalities (surgery, chemotherapy, radiotherapy), were not significantly different between the two groups.

### Risk of venous thromboembolism in cancer patients

FVL mutation was detected in 21 of 182 cases (11.5%) and in 4 of 182 control patients (2.2%) (p = 0.0004). Among the 25 participants with factor V Leiden mutation, only one case had a homozygous status and all other cases and controls carried a heterozygous mutation. FVL mutation was associated with a nearly seven-fold increased risk of VTE in cancer patients (crude OR, 6.67 [95% CI, 1.98–22.43], p = 0.002) (Table 2).

**Table 2:** Odds ratios for venous thromboembolism risk.

Variable	Univariate analysis, Crude odds ratio (95% CI)	p-value	Multivariate analysis, Adjusted* odds ratio (95% CI) Model 1	p-value
**Factor V Leiden mutation**	6.67 (1.98–22.43)	0.002	7.04 (2.01–24.63)	0.002
**Cancer Stage**, n (%)				
No distant metastasis	1 (Reference)		1 (Reference)	
Metastatic disease	1.29 (0.81–2.06)	0.29	1.49 (0.91–2.45)	0.11
Not classifiable	∞	1	∞	1
**Family history of VTE**	2.00 (0.97–4.12)	0.06	2.31 (1.02–5.26)	0.045

CI, confidence interval; VTE, venous thromboembolism

* 168 pairs

multivariate analysis adjusted on cancer stage and family history of VTE.

In main multivariate analysis adjusted for cancer stage and family history of venous thromboembolism (Table 2), we confirmed an odds ratio for VTE of 7.04 (95% CI, 2.01–24.63, p = 0.002) due to FVL mutation in cancer patients. In addition, the OR for VTE in cancer patients with family history of VTE was 2.31 (95% CI, 1.02–5.26, p = 0.045). In order to limit confounding risk factors, we performed a secondary multivariate analyses to evaluate the impact of FVL mutation on VTE, according to whether a familial or a personal history of VTE was present or not (Table 3); these analyses confirmed FVL as a significant risk factor for VTE in cancer patients.

**Table 3:** Odds ratio for venous thromboembolism risk according sub-groups.

Variables according to patients’ selection	Univariate analysis, Crude odds ratio (95% CI)	p-value	Multivariate analysis, Adjusted odds ratio (95% CI)	p-value
**Patients without family history of VTE (n = 157 pairs)**				
**Factor V Leiden mutation**	5.72 (1.62–20.16)	0.002	6.27 (1.39–28.23)	0.017
**Cancer Stage**, n (%)				
No distant metastasis	1 (Reference)		1 (Reference)	
Metastatic disease	1.14 (0.69–1.87)	0.61	1.21 (0.71–2.07)	0.48
Not classifiable	∞	1	∞	0.99
**Patients with family history of VTE (n = 14 pairs)**				
Factor V Leiden mutation*	-		-	
**Cancer Stage**, n (%)				
No distant metastasis	1 (Reference)		1 (Reference)	
Metastatic disease	6.51 (1.39–30.36)	0.02	∞	0.02
Not classifiable	∞	1	∞	1
**Patients without personal history of VTE (n = 149 pairs)**				
**Factor V Leiden mutation**	5.33 (1.55–18.29)	0.008	5.40 (1.54–18.95)	0.008
**Cancer Stage**, n (%)				
No distant metastasis	1 (Reference)		1 (Reference)	
Metastatic disease	1.07 (0.64–1.79)	0.79	1.12 (0.66–1.90)	0.66
Not classifiable	∞	1	∞	1
Patients with personal history of VTE**				
**Factor V Leiden mutation**	-		-	
**Cancer Stage**, n (%)				
No distant metastasis	-		-	
Metastatic disease	-		-	
Not classifiable	-		-	

CI, confidence interval; VTE, venous thromboembolism

*Odds ratio for Factor V Leiden was not calculated because there were no controls with Factor V Leiden.

**Odds ratio for Factor V Leiden and cancer were not calculated because there was no controls with a personal history of VTE; the 33 VTE patients could be matched with controls.

## Discussion

In this large case-control study, we found an independent significant seven-fold increased risk of VTE in association with FVL mutation in cancer patients.

Our findings are consistent with the results of a large recent European prospective observational study, the Vienna Cancer And Thrombosis Study (CATS) [13]. Pabinger *et al*. included and followed 982 cancer patients for a maximum period of 2 years, from which 72 (7.3%) were diagnosed with the FVL mutation. Ten of 72 (13.9%) cancer patients with FVL developed VTE whereas 69 of 910 (7.6%) without FVL, leading to probabilities for development of VTE after 6 months of 13% and 5.7% in Kaplan-Meier analysis, respectively. In multivariate analysis including age, sex, tumor type, stage, newly diagnosed *versus* recurrent disease and treatment modalities, the hazard ratio for VTE in cancer patients with FVL mutation was 2.0 (95% CI, 1.0–4.0). They concluded that FVL mutation was an independent risk factor of VTE in cancer patients, but the increase in the risk of VTE seemed to be lower than our findings, what can probably be partly explained by our case-control design that tends to overestimate the risk extent.

In the Multiple Environmental and Genetic Assessment (MEGA) study [5], a large population-based case-control study, the Factor V Leiden mutation was assessed in 2706 VTE cases and in 1757 control participants. Frequencies of the gene mutation were 8.1% and 2.8% in cases and controls respectively. There was a five-fold increased risk of VTE associated with cancer alone and the thrombotic risk due to FVL mutation was superimposed, with a twelve-fold increased risk as compared to individuals without cancer and FVL mutation (adjusted OR, 12.1 [95% CI, 1.6–88.1]). Finally, when only considering cancer patients, the adjusted OR for VTE due to FVL mutation was 2.2 (95% CI, 0.3–17.8), similarly to the data showed in the CATS prospective study [13]. Another case-control study including 101 pairs of cancer patients identified five cases and three controls with FV Leiden mutation [15]. In this study the risk of VTE associated with FVL mutation was close to the previous data (OR, 1.7 [95% CI, 0.3–10.7]) but was not significant due to the lack of power. A more recent case-control study including 124 cases, each matched with two controls, found a FVL mutation in 23 (18.5%) cases and 12 (4.8%) controls, yielding a five-fold increased risk of VTE (OR, 4.66 [95% CI, 2.14–10.14]) closer to our results, but in a selected population of breast cancer treated with tamoxifen [21]. A last case-cohort study including 609 cases in Norway investigated joint effects of cancer and variants in the Factor V gene on the risk of VTE [16]. A synergistic interaction was found between active cancer and FVL mutation with an excess in the relative risk due to interaction of 7.0 (95% CI, 0.5–14.4).

The G20210A variant of the prothrombin gene is another frequent mutation reported to be a risk factor for VTE [10] with a lower prevalence of around 2% [22], explaining our smaller number of patients, five cases and one control, which did not permit us to conclude about this parameter. A general review on prothrombin gene mutation and cancer found two case-control designed and a small cohort study concluding to an increased risk of VTE due to this mutation in cancer patients, but all with insufficient power due to limited numbers of patients resulting in wide confidence intervals for risk estimates [23]. A more recent cohort of 381 patients with breast and digestive cancer receiving adjuvant chemotherapy could not show any association between the prothrombin G20210A mutation and a risk of VTE [24].

Whereas it is admitted in the literature that the presence of metastases increases the thrombotic risk still further [13,23,25], our results did not confirm this observation. We only found a trend towards more metastatic status in cases and more locoregional status in controls (p = 0.08), probably because of a lack of power. Similarly, anti-neoplastic therapy is often associated with a hypercoagulable state [26, 27] but our results did not reveal any influence of the cancer treatment on the thrombotic risk, matching with the CATS study findings [13]. To go further we assessed another risk factor for cancer-associated thrombosis that is histology. For several authors, the risk of VTE is particularly high in patients with adenocarcinomas [27,28] but this was not verified in the present analysis of the EDITH study.

The main limitation of our study is related to its case-control design from which we cannot directly infer absolute risks or derive statements about therapeutic strategies. Thus, because of the small prevalence of FVL mutation, this design was adapted to answer the question of an increased risk of VTE in cancer patients carrying the mutation. Moreover, to the best of our knowledge, this study currently includes the largest number of cases and controls to assess the association between FVL mutation and cancer-associated thrombosis, with a total of 364 participants, allowing us to bring conclusions having solid intern validity.

Another limitation was the high number of cases with no eligible control (n = 311) that has the potential to create a selection bias with a risk of over- or underestimation of the increased risk of VTE in association with FVL mutation in cancer patients. Particularly, this might explain the low prevalence of FVL in our controls. However, our sample size calculation indicated that 150 pairs were needed to detect a 4-fold increase in VTE risk and we succeeded to match 182 pairs. We should then mention a potential bias due to the fact that we had a tendency to more cases with familial history of VTE than controls (13.7 vs. 7.7%, p = 0.08), that could have lead us to overestimate the impact of FVL in the increase of VTE risk. To minimize this bias, we conducted a second multivariate analysis model on 134 pairs of patients excluding pairs with family history of VTE, and we confirmed a six-fold increase of cancer-associated thrombosis risk due to FVL mutation (adjusted OR, 6.27 [1.39–28.23], p = 0.017). Similarly, a personal past history of VTE was an exclusion criteria for control subjects but not for cases, leading us to a significant difference in the two groups with 18.1% of cases having a past thrombosis history. This could also have led us to overestimate the increase of the VTE risk associated with the factor V Leiden mutation in cancer patients. For this reason, we performed an additional multivariate analysis, excluding cases with a personal past history of VTE, which confirmed a nearly six-fold increased risk (adjusted OR, 5.40 [95% CI, 1.54–18.95]). In comparison, the case-control MEGA study, which included only participants having a first-episode of venous thrombosis, confirmed an increase in the risk of VTE in association with FVL in cancer patients, but only by a factor two (adjusted OR, 2.2 [95% CI, 0.3–17.8]) [5], similarly to the CATS study [13].

Regarding this additional observation of the high risk of VTE in patients associating cancer and FVL mutation, our results suggest that this thrombophilia might be taken into account in the individual thrombosis risk assessment. However, even if FVL mutation test is widely available and has excellent sensitivity and specificity, these results cannot warrant routine screening of all cancer patients to identify a small number of patients overly predisposed for VTE. Moreover, the impact of FVL screening in terms of thrombosis prophylaxis with anticoagulant therapy such as low-molecular weight heparin has never been tested in patients with cancer-associated thrombosis carrying FVL mutation, as cancer patients have an increased risk of thrombosis and bleeding. In the MEGA study, Blom *et al*. demonstrated that screening for FVL and the prothrombin 20210A gene mutation in cancer patients, assuming that subsequent prophylactic anticoagulant therapy would be effective for 80% of them, would prevent annually only 7 to 27 venous thrombotic events per 10,000 cancer patients screened [5]. Universal screening of cancer patients for pro-thrombotic mutations seems thus neither useful, nor cost-effective.

In conclusion, we found that Factor V Leiden mutation was an independent additional risk factor for venous thromboembolism in cancer patients. Whether screening for such inherited thrombophilia might have an impact on clinical practice requires prospective validation.
